# Differential Responses of Flowering Functional Groups to Species Loss Altered Community Flowering-Date Synchrony and Reduced Temporal Stability in an Alpine Meadow

**DOI:** 10.3390/plants15182822

**Published:** 2026-09-14

**Authors:** Pingyu Liu, Shiqi Xiong, Jun Chen, Wenzhang Dai

**Affiliations:** 1 School of Life Sciences, Nanjing University, Nanjing 210023, China; dg21300070@smail.nju.edu.cn (P.L.);; 2 School of Ecology and Environment, Key Laboratory of Biodiversity and Environment on the Qinghai-Tibet Plateau, Ministry of Education, Xizang University, Lhasa 850000, China

**Keywords:** species-specific plant phenology, species removal, flowering phenology, interspecific interactions, alpine meadow

## Abstract

Understanding how biodiversity loss alters flowering patterns in plant communities is crucial for predicting species coexistence, community dynamics, and ecosystem functioning under global change. Using phenological records from 2017 to 2023 within a 12-year species removal experiment in a Tibetan alpine meadow, we examined responses of early-, mid-, and late-flowering functional groups and changes in community flowering-date synchrony and temporal stability. Species removal tended to advance first flowering but more consistently delayed last flowering and extended flowering duration, with differential responses among flowering functional groups leading to contrasting changes in community flowering-date synchrony: forb removal decreased synchrony, whereas grass and dominant-sedge removal increased synchrony. Forb removal, grass removal, and proportional thinning also reduced the temporal stability of community first and last flowering dates. Declining species richness and lower richness stability were associated with increased phenological divergence among flowering functional groups, which predicted 88% and 85% of the variation in community-wide dispersion of first and last flowering dates, respectively. Greater dispersion was associated with lower temporal stability only for first flowering. These findings suggest a temporal-niche pathway through which species loss may reorganize community flowering phenology and help predict long-term changes in floral resource availability and ecosystem functioning in alpine meadows.

## 1. Introduction

Understanding how plant phenology responds to global change is essential for predicting species coexistence, community dynamics, and ecosystem functioning [1,2,3]. Flowering times are often strongly differentiated among species within plant communities, producing a seasonal sequence composed of early-, mid-, and late-flowering functional groups [4,5]. Such phenological differentiation not only reflects differences in species life-history strategies, but also represents an important arrangement of temporal niches within communities [6,7]. As global change intensifies, ongoing plant species loss is altering community composition, interspecific interactions, and patterns of resource allocation, potentially disrupting established patterns of phenological differentiation and overlap and thereby reorganizing the temporal structure of community flowering [8,9]. Earlier research focused primarily on the effects of abiotic drivers, including temperature, precipitation, and nutrient availability, and showed that first flowering date, last flowering date, and flowering duration can respond in different directions and to different magnitudes among species and plant functional groups [10,11,12]. More recent work has further demonstrated that divergent responses among flowering functional groups can alter community flowering-date synchrony and the length of the flowering season [4,13]. However, the role of plant species loss as an important biotic driver in shaping the relative phenological responses of early-, mid-, and late-flowering functional groups, and consequently community flowering-date synchrony and its temporal stability, remains poorly understood [2,8,14].

Previous studies have shown that declines in plant diversity can alter the competitive and microenvironmental conditions experienced by the remaining species, potentially inducing phenological shifts. Earlier studies showed that plant diversity loss can advance flowering in many species and alter the temporal distribution of flowering events within communities through changes in near-surface soil temperature, soil nitrogen availability, and soil moisture [8,15]. Recent evidence further shows that plant species loss generally advances the first flowering dates and delays the last flowering dates of the remaining species, thereby extending their flowering durations [9]. However, most studies have focused on mean community flowering time or on the phenological responses of a subset of species, with relatively little attention to whether early-, mid-, and late-flowering functional groups respond differently. These three flowering functional groups occupy the beginning, peak, and end of the community flowering season, respectively, and shifts in their relative phenological positions directly affect interspecific flowering overlap and the length of the community flowering season [4,7]. Distinguishing among the responses of early-, mid-, and late-flowering functional groups is therefore important for understanding how species loss reorganizes community flowering phenology from a temporal-niche perspective.

Because three flowering functional groups occupy different portions of the growing season, they may be regulated by different environmental and biotic processes. Early-flowering functional groups may be more strongly influenced by thermal accumulation and shallow-soil resource conditions early in the growing season, mid-flowering groups may be more sensitive to water supply, community density, and competitive intensity during the period of rapid community growth, and late-flowering groups may be more constrained by late-season resource conditions and community structure [12,16,17,18]. Changes in environmental conditions and community structure caused by species loss may therefore elicit distinct phenological responses among early-, mid-, and late-flowering functional groups, with the direction and magnitude of these responses also depending on the functional attributes of the lost and remaining species [8,19]. Differential phenological responses among flowering functional groups may further influence community flowering-date synchrony and temporal stability [13,20]. Flowering synchrony describes the temporal overlap of flowering among species and provides an important link between species-level phenological shifts and community-level temporal structure [21,22]. If early-, mid-, and late-flowering functional groups shift in similar directions and by similar magnitudes following species loss, temporal distances among species may remain relatively stable and community flowering-date synchrony may change little. Conversely, if one flowering functional group is more responsive than the others, temporal distances among species may expand or contract, reducing or increasing flowering synchrony. Lower synchrony indicates a more dispersed distribution of flowering events, which may reduce flowering overlap and competition for pollinators but may also weaken the collective attraction of co-flowering species. Higher flowering synchrony concentrates the flowering of multiple species within a shorter time window and may therefore alter pollinator-mediated interactions among plants [23,24]. Importantly, changes in community flowering-date synchrony may also affect the temporal stability of community phenology [25,26]. At high species diversity, temporal complementarity and asynchronous responses among flowering functional groups may buffer interannual climatic variation, thereby stabilizing community flowering dates, flowering-season length, and the supply of reproductive resources [25,27]. Species loss may weaken this temporal complementarity, increase the dependence of community phenology on a small number of functional groups or dominant species, amplify interannual variability, and thereby reduce temporal stability [25,26,27]. Therefore, species loss may alter not only the flowering times of individual species, but also the relative phenological relationships among early-, mid-, and late-flowering functional groups, with consequences for flowering synchrony and the temporal stability of alpine meadow phenology.

**Figure 1:**
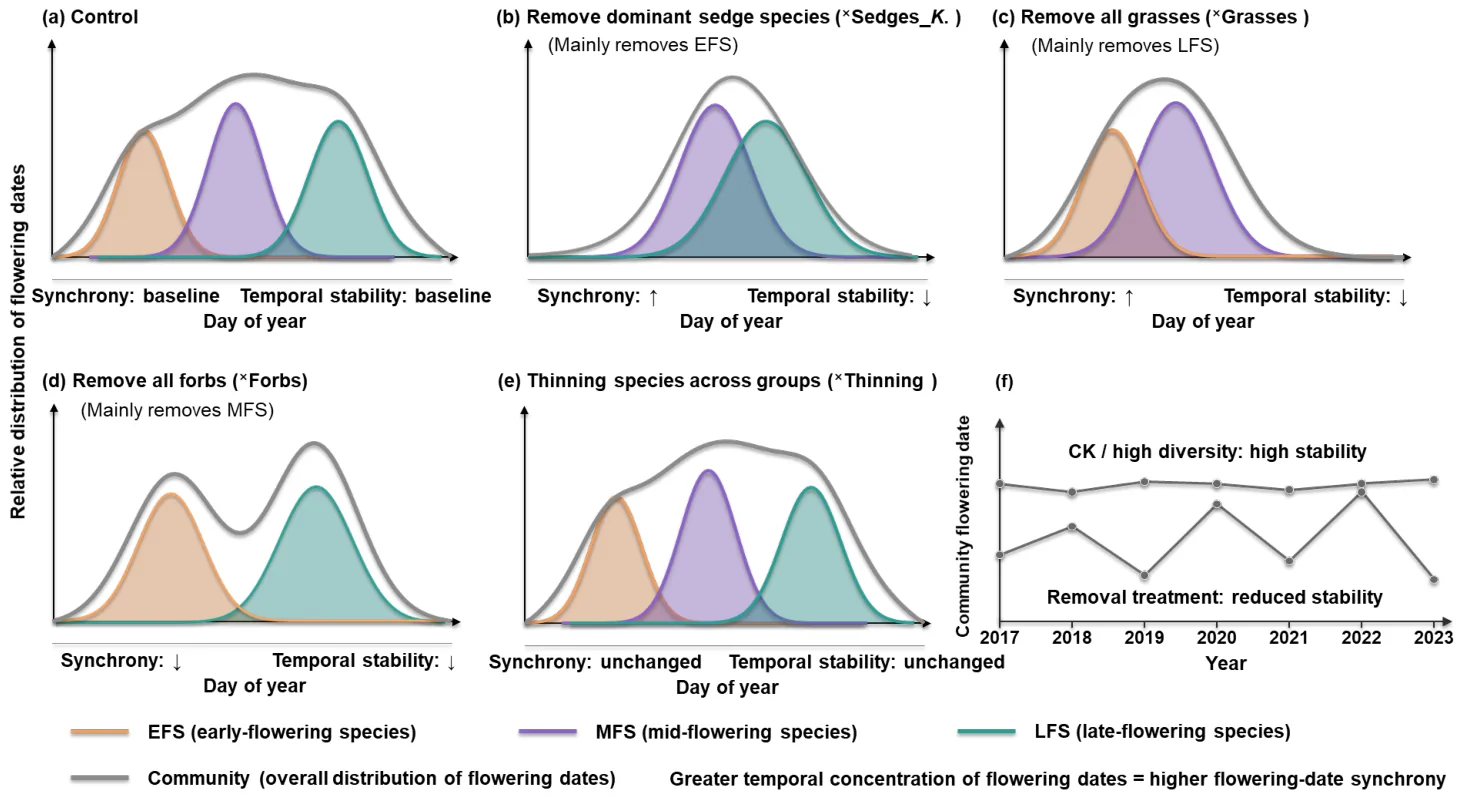
Conceptual framework illustrating how removal of different plant functional groups may affect community flowering-date synchrony and temporal stability. (**a**) In the control community, early-flowering (EFS), mid-flowering (MFS), and late-flowering (LFS) functional groups occupy complementary phenological niches and maintain baseline levels of synchrony and temporal stability. (**b**) Removal of the early-flowering dominant sedge is expected to increase flowering overlap and synchrony but reduce temporal stability. (**c**) Removal of late-flowering grasses is expected to increase flowering synchrony but reduce temporal stability. (**d**) Removal of mid-flowering forbs is expected to reduce flowering overlap between the early- and late-flowering functional groups, thereby decreasing synchrony and temporal stability. (**e**) Proportional thinning across plant functional groups is expected to preserve overall flowering structure and therefore have relatively weak effects on synchrony and temporal stability. Colored curves schematically represent the temporal distribution of flowering dates within the three flowering functional groups, and the grey curve represents the overall community distribution. Greater temporal concentration of flowering dates corresponds to higher flowering-date synchrony, whereas greater dispersion corresponds to lower synchrony. (**f**) Communities that retain diverse and complementary flowering functional groups are expected to have greater temporal stability, whereas species loss weakens community buffering capacity and reduces stability.

Accordingly, drawing on data from a long-term species removal experiment in an alpine meadow on the Tibetan Plateau, we examined the phenological responses of early-, mid-, and late-flowering functional groups to different removal treatments and further assessed changes in community flowering-date synchrony and temporal stability. We predicted that species loss would reorganize community flowering structure by altering the response magnitude and relative phenological positions of flowering functional groups. In the unmanipulated control (Control), early-, mid-, and late-flowering functional groups occupy complementary phenological niches, maintaining a relatively continuous flowering season and buffering interannual variation (Figure 1a,f). Following removal of the early-flowering dominant sedge (^×^Sedges_*K*.) or late-flowering grasses (^×^Grasses), competitive release was expected to advance first flowering, delay last flowering, and increase flowering overlap among the remaining species, thereby increasing synchrony. However, the loss of early- or late-season flowering components was expected to reduce the temporal stability of community flowering phenology (Figure 1b,c,f). Removal of mid-flowering forbs (^×^Forbs) was expected to weaken temporal continuity between the early- and late-flowering functional groups and consequently reduce both flowering synchrony and temporal stability (Figure 1d,f). By contrast, proportional thinning across plant functional groups (^×^Thinning) was expected to better preserve the relative structure of the early-, mid-, and late-flowering functional groups and thus have weaker effects on synchrony and temporal stability (Figure 1e).

## 2. Results

### 2.1. Species Loss Elicited Divergent Responses Among Flowering Functional Groups

**Figure 2:**
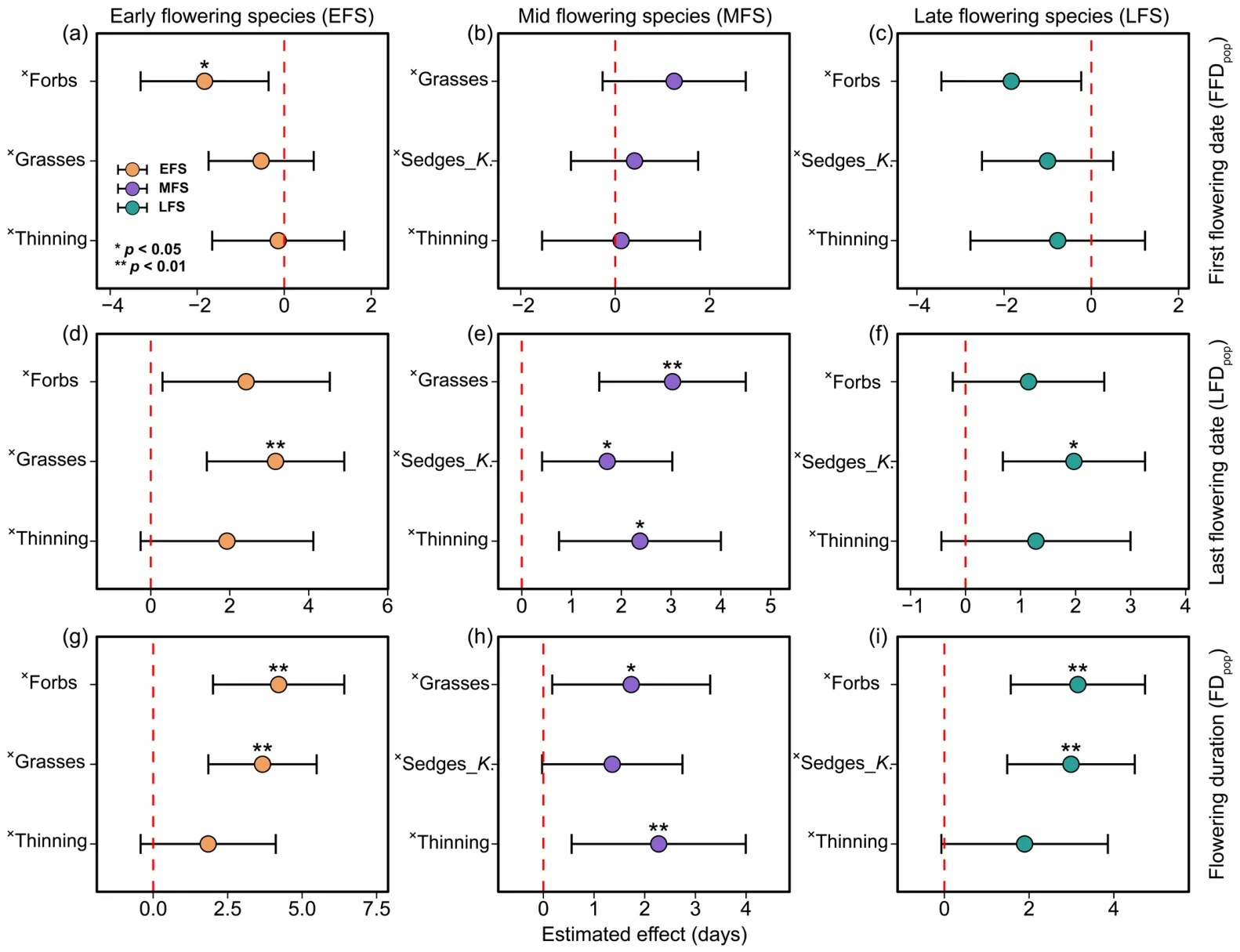
Effects of removal treatments on first flowering date (FFD_pop_; (**a**–**c**)), last flowering date (LFD_pop_; (**d**–**f**)), and flowering duration (FD_pop_; (**g**–**i**)) of the early-, mid-, and late-flowering functional groups. Phenological responses of the different flowering functional groups are presented as the estimated effects of each removal treatment relative to the control (estimate ± 95% CI). Colors denote flowering functional groups: orange, early- (EFS); purple, mid- (MFS); and blue-green, late- (LFS) flowering functional groups. Negative values indicate earlier first or last flowering, or shorter flowering duration, relative to the control; positive values indicate later first or last flowering, or longer flowering duration. Asterisks indicate statistical significance based on Benjamini–Hochberg-adjusted *p*-values across the 27 treatment comparisons: * adjusted *p* < 0.05; ** adjusted *p* < 0.01.

Differences in phenological responses among the three flowering functional groups were most pronounced for last flowering date (LFD_pop_) and flowering duration (FD_pop_), whereas their responses in first flowering date (FFD_pop_) were limited (Figure 2a–c). Specifically, the early-flowering functional group showed significant responses to multiple removal treatments. Forb removal significantly advanced FFD_pop_ by 1.9 ± 0.8 days and extended FD_pop_ by 4.1 ± 1.1 days. Grass removal significantly delayed LFD_pop_ by 3.2 ± 0.9 days and extended FD_pop_ by 3.7 ± 0.9 days. Proportional thinning did not significantly affect these phenological variables. Responses of the mid-flowering functional group were expressed mainly as delayed LFD_pop_. Grass removal, dominant-sedge removal, and proportional thinning resulted in significant delays in LFD_pop_ of 3.0 ± 0.8, 1.7 ± 0.7, and 2.4 ± 0.8 days, respectively. Grass removal and proportional thinning also significantly extended FD_pop_ by 1.7 ± 0.8 and 2.3 ± 0.9 days, whereas the increase following dominant-sedge removal was not significant. The late-flowering functional group responded significantly only to forb removal and dominant-sedge removal. Forb removal advanced its FFD_pop_ by 1.8 ± 0.8 days and significantly extended FD_pop_ by 3.1 ± 0.8 days, whereas dominant-sedge removal significantly delayed LFD_pop_ by 2.0 ± 0.7 days and consequently extended FD_pop_ by 3.0 ± 0.8 days (Figure 2d–i). Thus, species removal did not induce uniform phenological responses across flowering functional groups; instead, the clearest effects were observed for LFD_pop_ and flowering duration, with responses differing markedly in direction and magnitude among flowering functional groups.

### 2.2. Species Removal Non-Randomly Altered Community Flowering-Date Synchrony

**Figure 3:**
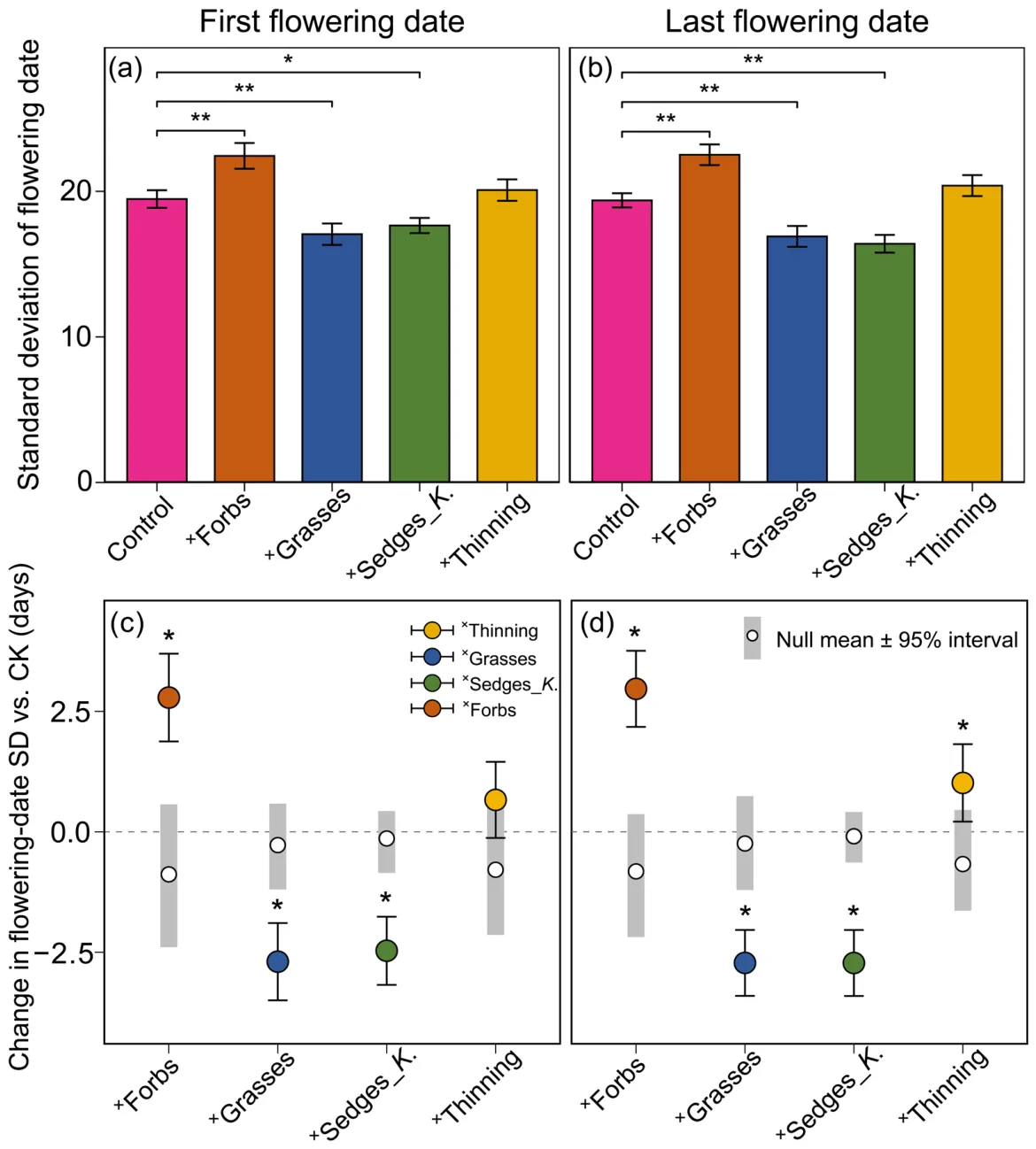
Effects of species removal treatments on community flowering-date synchrony (**a**,**b**) and their deviation from expectations under random species loss (**c**,**d**). In panels (**a**,**b**), community flowering-date synchrony is represented by the standard deviation of first flowering dates (FFD_sd) and last flowering dates (LFD_sd) among species within each treatment; larger standard deviations indicate greater temporal dispersion and lower synchrony. Bars and error bars show means and standard errors, respectively. Asterisks indicate significant differences from control: * *p* < 0.05; ** *p* < 0.01. Panels (**c**,**d**) show treatment-induced changes in the standard deviation of first and last flowering dates relative to control and compare observed removal effects with the random species-loss null model. Colored filled circles and error bars show the mean and standard error of the observed removal effects; open circles and grey vertical bars show the null-model mean and 95% randomization interval, respectively. Positive and negative values indicate decreased and increased synchrony, respectively. Asterisks indicate that the observed removal effect deviated significantly from the null-model expectation.

Species removal treatments significantly altered community flowering-date synchrony in both first and last flowering dates, with the same treatments producing significant effects on both metrics. Community flowering-date synchrony was inversely represented by the among-species standard deviation of flowering dates: larger standard deviations indicate lower synchrony and greater flowering-date dispersion, whereas smaller standard deviations indicate higher synchrony and lower dispersion (Figure 3a,b). Forb removal significantly increased the standard deviation, indicating lower community flowering-date synchrony. By contrast, grass removal and dominant-sedge removal significantly reduced the standard deviation, indicating that flowering dates of the remaining species became more concentrated and community synchrony increased. Proportional thinning had a comparatively weak effect and did not differ significantly from the control. Comparison with the random species-loss null model showed that the increase in standard deviation following forb removal and the decreases following grass and dominant-sedge removal all deviated significantly from random expectations, indicating that their effects on community flowering-date synchrony were strongly non-random (Figure 3c,d). Proportional thinning deviated significantly from the null model only for the standard deviation of last flowering date, whereas its effect on first flowering date remained within random expectations, further indicating an overall weak effect on community flowering-date synchrony.

### 2.3. Species Loss Altered Interannual Trajectories and Reduced the Temporal Stability of Community Flowering Phenology

**Figure 4:**
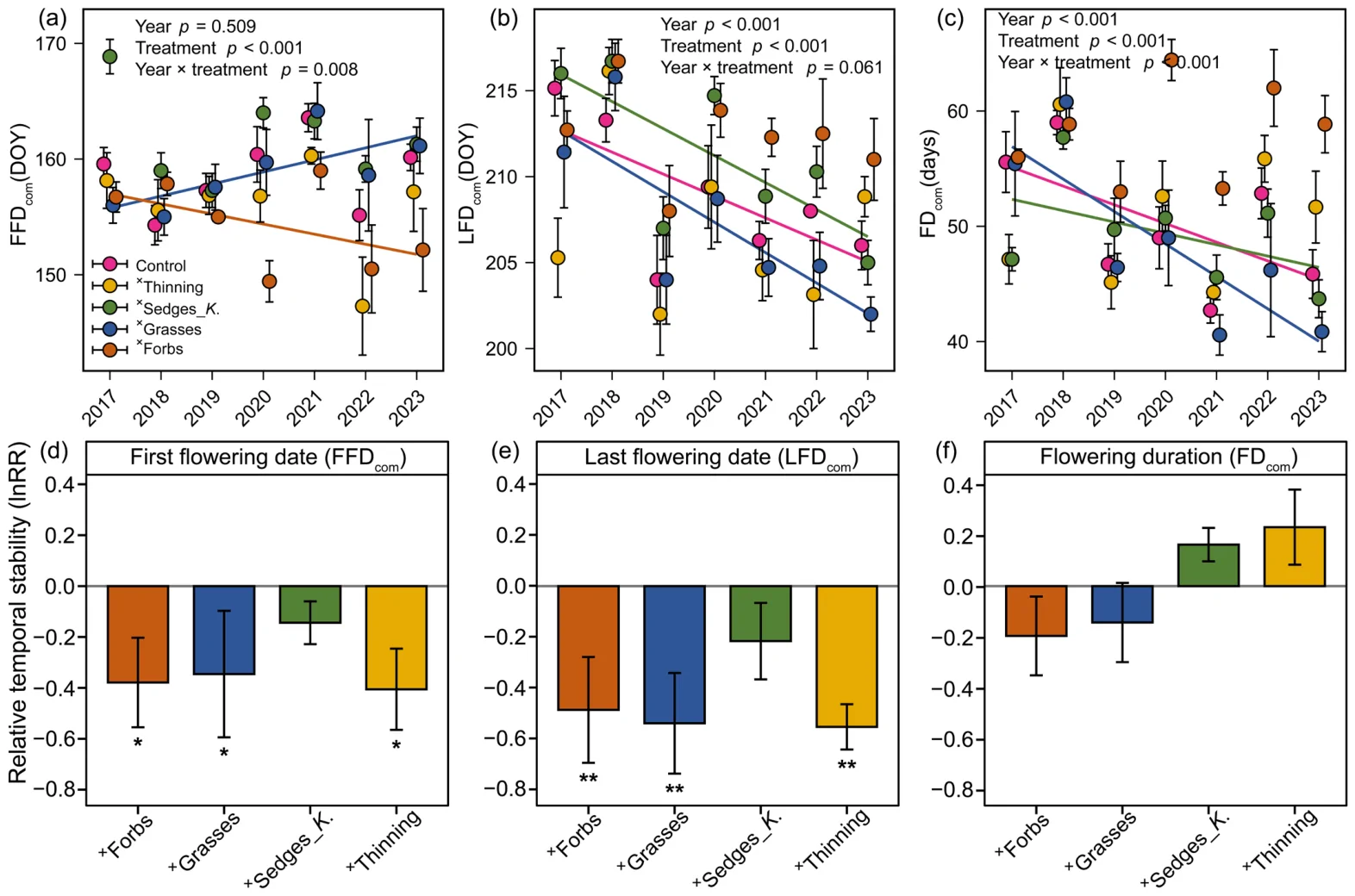
Effects of species loss on interannual dynamics (**a**–**c**) and temporal stability (**d**–**f**) of community flowering phenology. Interannual changes in community first flowering date (FFD_com_), last flowering date (LFD_com_), and flowering duration (FD_com_) under the species-removal treatments from 2017 to 2023 are shown in panels (**a**–**c**). Points and error bars represent means ± SE, and solid lines are shown only for treatment-specific linear trends that were significant at *p* < 0.05. Test statistics for the effects of year, treatment, and their interaction are shown in each panel. Panels (**d**–**f**) show treatment-induced changes in the temporal stability of FFD_com_, LFD_com_, and FD_com_ relative to the control, expressed as the log response ratio lnRR [ln(stability_treatment_/stability_Control_)]. Values of lnRR < 0 indicate lower temporal stability under the removal treatment than in the control, whereas lnRR > 0 indicates higher temporal stability. Asterisks indicate that lnRR differed significantly from zero and thus that temporal stability differed between the removal treatment and control: * *p* < 0.05; ** *p* < 0.01.

From 2017 to 2023, community-level first flowering date (FFD_com_) and last flowering date (LFD_com_) exhibited pronounced interannual dynamics, and species loss altered both the direction and magnitude of these temporal trends. FFD_com_ showed a significant treatment effect (F = 11.50, *p* < 0.001) and year × treatment interaction (F = 3.54, *p* = 0.008), whereas the main effect of year was not significant (F = 0.44, *p* = 0.509), indicating that interannual trends in FFD_com_ differed among treatments. Under grass removal, FFD_com_ was significantly delayed at a rate of 1.08 ± 0.45 d yr^−1^ (*p* = 0.017). No significant interannual trends were detected under the other treatments, although forb removal produced a marginal advance (−0.84 ± 0.43 d yr^−1^, *p* = 0.053; Figure 4a). LFDcom showed a strong overall advance over time. Both year (F = 35.14, *p* < 0.001) and treatment (F = 9.15, *p* < 0.001) had significant effects, whereas the year × treatment interaction was not significant (F = 2.29, *p* = 0.061). LFD_com_ advanced significantly under the control, dominant-sedge removal, and grass-removal treatments at rates of −1.28 ± 0.41, −1.57 ± 0.41, and −1.69 ± 0.43 d yr^−1^, respectively; no significant trend was detected under forb removal or proportional thinning across functional groups (Figure 4b). FDcom was significantly affected by year, treatment, and their interaction (year: F = 17.36, *p* < 0.001; treatment: F = 14.45, *p* < 0.001; year × treatment: F = 6.57, *p* < 0.001). FD_com_ was significantly shortened under the control and grass-removal treatments at rates of −1.62 ± 0.51 and −2.77 ± 0.54 d yr^−1^, respectively. It also tended to decline under dominant-sedge removal, although this trend was not significant (−0.98 ± 0.51 d yr^−1^, *p* = 0.056); no clear interannual trends occurred under the remaining treatments (Figure 4c). In terms of temporal stability, forb removal, grass removal, and proportional thinning across functional groups significantly reduced the relative temporal stability of both FFD_com_ and LFD_com_, whereas dominant-sedge removal had no significant effect (Figure 4d,e). The temporal stability of FD_com_ was not significantly affected by any species-removal treatment (Figure 4f).

### 2.4. Effects of Species Richness and Soil Environmental Change on Phenological Divergence Among Flowering Functional Groups, Community Synchrony, and Temporal Stability

**Figure 5:**
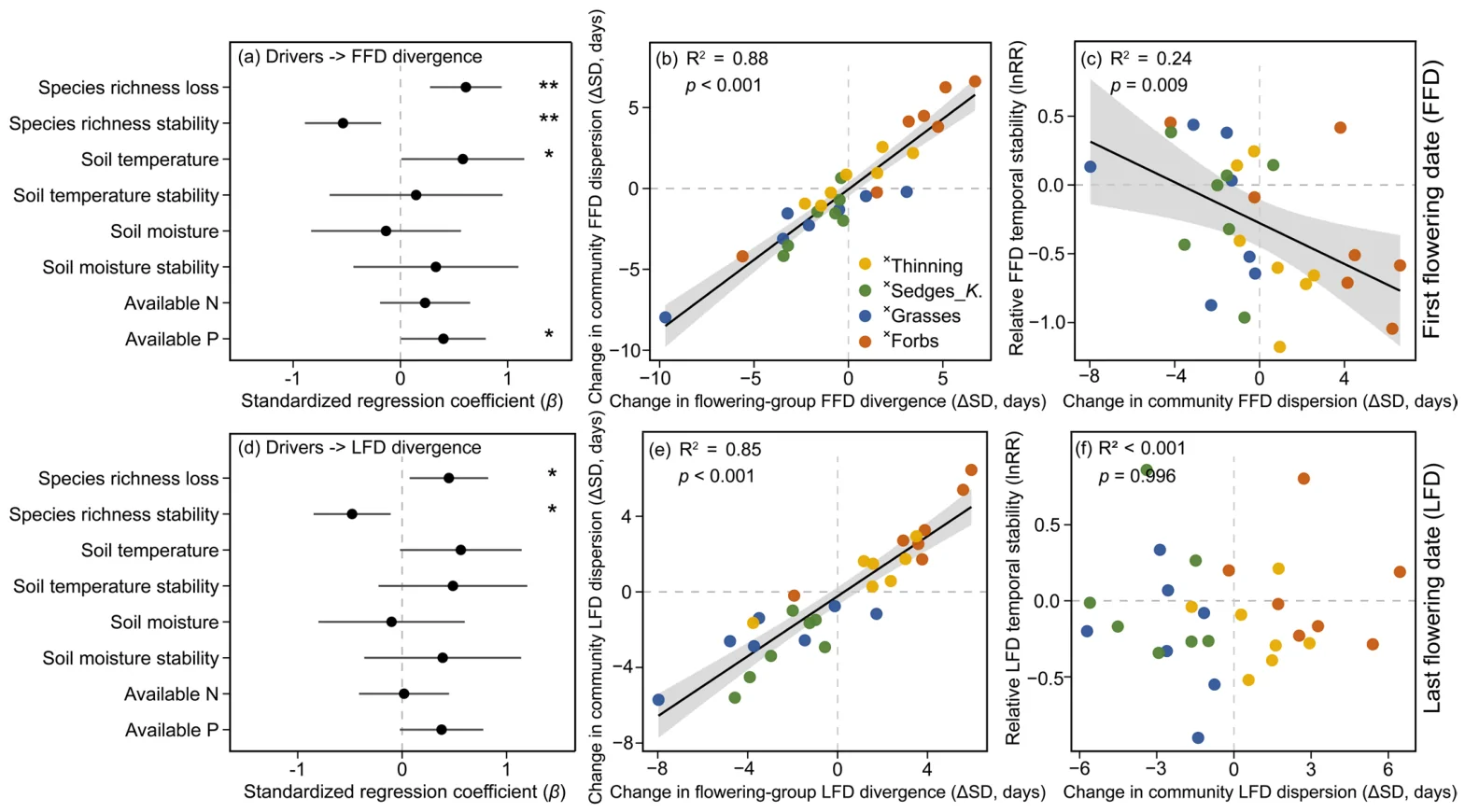
Relationships between species richness and soil environmental changes with phenological divergence among flowering functional groups (**a**,**d**), phenological divergence with community-wide flowering-date dispersion (**b**,**e**) and flowering-date dispersion with temporal stability (**c**,**f**). Phenological divergence in first flowering date (FFD) and last flowering date (LFD) was quantified as the standard deviation of the mean flowering dates of the early-, mid-, and late-flowering functional groups. Panels (**a**,**d**) show standardized regression coefficients (*β*) and their 95% confidence intervals for the effects of each driver on phenological divergence. Asterisks indicate coefficients significantly different from zero: * *p* < 0.05; ** *p* < 0.01. Because flowering-date dispersion is inversely related to flowering-date synchrony, positive ΔSD values indicate reduced synchrony, whereas negative ΔSD values indicate increased synchrony. Relative temporal stability was expressed as lnRR = ln(stability_treatment_/stability_Control_), with lnRR < 0 indicating lower stability than control and lnRR > 0 indicating higher stability. Points represent treatment × block combinations; solid lines and grey shading show significant linear regressions and their 95% confidence intervals. Changes in phenological divergence explained 88% (R^2^ = 0.88, *p* < 0.001) and 85% (R^2^ = 0.85, *p* < 0.001) of the changes in community-wide FFD and LFD dispersion, respectively. Changes in FFD dispersion explained 24% of the variation in relative temporal stability (R^2^ = 0.24, *p* = 0.009), whereas LFD dispersion was not significantly related to temporal stability.

Multiple regression analyses showed that phenological divergence among flowering functional groups in first flowering date increased with declining species richness, increasing soil temperature, and increasing soil available phosphorus, but decreased with greater temporal stability of species richness; no other soil variable had a significant effect (Figure 5a). Divergence in last flowering date was driven primarily by changes in species richness. Lower species richness significantly increased the temporal separation among early-, mid-, and late-flowering functional groups, whereas greater temporal stability of species richness significantly reduced this separation; none of the soil environmental variables had a significant effect (Figure 5d). Thus, declining species richness and greater interannual variability in richness jointly promoted phenological divergence among flowering functional groups.

Phenological divergence among flowering functional groups was positively related to community-wide dispersion of both first and last flowering dates and explained 88% and 85% of the variation in their respective changes (both *p* < 0.001; Figure 5b,e). This result indicates that greater temporal separation among early-, mid-, and late-flowering functional groups disperses flowering dates across the community and thereby reduces community flowering-date synchrony. Increased dispersion of first flowering date was significantly associated with reduced temporal stability (R^2^ = 0.24, *p* = 0.009; Figure 5c), whereas dispersion of last flowering date was unrelated to its temporal stability (R^2^ < 0.001, *p* = 0.996; Figure 5f). Overall, declining species richness and reduced temporal stability of richness promoted phenological divergence among flowering functional groups, while higher soil temperature and greater soil available phosphorus further increased divergence in first flowering date. Greater phenological divergence among flowering functional groups was closely associated with lower community flowering-date synchrony, but the link between reduced synchrony and reduced temporal stability was supported only for first flowering date.

## 3. Discussion

Predicting how flowering phenology responds to global change is essential for understanding community dynamics and ecosystem stability. However, how plant species loss alters the phenological responses of different flowering functional groups remains poorly understood. Overall, our results partially supported our hypotheses. In this alpine meadow, species loss reorganized community flowering phenology through divergent responses among early-, mid-, and late-flowering groups, primarily by delaying last flowering and extending flowering duration, whereas effects on first flowering were generally weak. These asymmetric responses altered community flowering-date synchrony, but the direction of change depended on the identity and phenological position of the removed species. Species loss also reduced the temporal stability of community first and last flowering dates, but not that of flowering duration.

### 3.1. Species Loss Altered Community Flowering-Date Synchrony Through Divergent Responses Among Flowering Functional Groups

Species loss had generally weak effects on first flowering date across flowering functional groups, with significant advances occurring only in the early-flowering groups following forb removal. Although statistically supported, the absolute shifts were modest (generally about 1–4 days). By contrast, significant responses in last flowering date were detected more frequently across flowering functional groups and removal treatments, often accompanied by corresponding changes in flowering duration. Species loss therefore appeared more likely to alter how long plants maintained reproductive activity than when they entered the reproductive phase [8,28]. This may be because first flowering date is often strongly constrained by early-season temperature, snowmelt timing, developmental progression, and other phenological cues [2,29], whereas last flowering date is more responsive to resource availability and neighborhood competition during the growing season [13,30]. Species removal may affect flowering phenology through multiple pathways, including both competitive release and changes in local microenvironmental conditions. By reducing canopy shading, species removal may increase light availability and alter soil temperature, moisture, and nutrient availability. Our supplementary analyses showed that soil temperature was mainly associated with first and last flowering in the mid-flowering group, whereas soil available phosphorus was associated with phenological responses in the early- and late-flowering groups (Figure S1). These results suggest that the asymmetric responses among flowering functional groups may reflect the combined effects of reduced competition and group-specific sensitivities to removal-induced microenvironmental changes.

Within the early-flowering functional group, forb removal advanced first flowering date and extended flowering duration, whereas grass removal delayed last flowering date and extended flowering duration. Forbs are species-rich, and their growth and flowering overlapped substantially with those of the early-flowering group during the early and middle portions of the growing season. Forb removal may have reduced resource competition before flowering, thereby allowing early-flowering species to initiate flowering earlier and maintain flowering for a longer period [6,7,31]. Supplementary analyses further showed that lower species richness was associated with earlier first flowering date and longer flowering duration in the early-flowering group (Figure S1a,g), whereas higher soil available phosphorus was associated with earlier last flowering date and shorter flowering duration (Figure S1f,i). Together, these relationships suggest that the response of the early-flowering group may reflect both competitive release following species loss and changes in soil available phosphorus. By contrast, grasses reached peak growth later in the season and therefore had limited effects on flowering initiation by early-flowering species. Their removal may instead have reduced competition during the later stages of flowering, allowing early-flowering species to remain in flower for longer.

First flowering date in the mid-flowering functional group did not change significantly under any treatment, whereas last flowering date was significantly delayed following grass removal, dominant-sedge removal, and proportional thinning. Flowering duration was significantly extended only following proportional thinning. The reproductive period of the mid-flowering group coincides with rapid canopy development and high community resource demand, and these species may therefore experience strong competition for light and below-ground resources [31,32]. Removal of the dominant sedge or grasses likely reduced both above- and below-ground competition and increased resource acquisition by the remaining mid-flowering species during the later stages of reproduction. Lower species richness was likewise associated with delayed last flowering date and longer flowering duration in the mid-flowering group (Figure S1d,g), supporting a role for competitive release in extending reproductive activity. Soil temperature was also positively associated with both first and last flowering dates in this group (Figure S1b,e), suggesting that thermal conditions may contribute to shifting its flowering window later. In the late-flowering functional group, forb removal advanced first flowering date, whereas dominant-sedge removal primarily delayed last flowering date; both responses extended flowering duration. Lower species richness was associated with earlier first flowering and longer flowering duration in the late-flowering group (Figure S1a,g). Thus, the longer flowering duration in this group may reflect both weakened competitive constraints following species richness loss and changes in soil available phosphorus (Figure S1i).

These asymmetric responses among flowering functional groups provide the mechanistic basis for changes in community flowering-date synchrony. Previous studies in alpine meadows have shown that flowering functional groups differ in the direction and magnitude of their responses to warming or nitrogen addition, thereby reducing or increasing community reproductive synchrony through the expansion or contraction of phenological distances among groups. Changes in the composition and relative cover of flowering functional groups can also modify community phenological responses to environmental change [4,13]. In the long-term species-removal experiment, forb removal reduced the number of species occupying the mid-season flowering window and created a gap in the otherwise continuous early–mid–late flowering sequence. Together with the divergent responses of the early- and late-flowering groups, this increased community-wide dispersion of flowering dates and reduced flowering synchrony. By contrast, grass removal and dominant-sedge removal reduced components at the end and beginning of the community flowering sequence, respectively, causing the phenology of the remaining species to shift in more similar directions and the distribution of flowering dates to become more concentrated, thereby increasing community flowering-date synchrony [6,33]. Proportional thinning better preserved the relative structure of the early-, mid-, and late-flowering functional groups and therefore had only weak effects on synchrony [7,13]. The null-model analysis further showed that changes in synchrony following forb, grass, and dominant-sedge removal all deviated significantly from random expectations. Community flowering-date synchrony was therefore determined not simply by the number of species lost, but also by the phenological position of those species and their interactions with the remaining flowering functional groups.

### 3.2. Species Loss Altered Interannual Trends in Community First and Last Flowering Dates and Reduced Temporal Stability

The direction and magnitude of interannual trends in FFD_com_ differed among removal treatments. FFD_com_ was progressively delayed under grass removal, tended to advance under forb removal, and changed relatively little under the other treatments. Even under identical changes in snowmelt, temperature, or soil moisture, species can differ in both the direction and magnitude of their flowering responses. Such species-specific responses can accumulate at the community level through changes in relative abundance and community composition and thereby reorganize community phenology [4,34]. FFD_com_ reflects not only the phenological responses of early-flowering species, but also changes in their relative abundance and in overall species composition. The tendency for FFD_com_ to advance under forb removal may therefore reflect the substantial reduction of the mid-flowering group and the increased relative contribution of early-flowering species. Conversely, the progressive delay under grass removal may reflect compensatory increases in mid-flowering forbs, which reduced the contribution of the earliest-flowering components to the onset of community flowering. In contrast to FFD_com_, LFD_com_ advanced significantly over time in the control, dominant-sedge removal, and grass-removal treatments, and the year × treatment interaction was not significant. Thus, LFD_com_ showed a broadly similar advance among treatments rather than a response unique to a particular removal treatment. Last flowering in alpine and montane plants is jointly regulated by temperature, snowmelt timing, and growing-season water availability [29,35]. Higher temperatures can accelerate phenological development and shift both first and last flowering dates earlier, while differences in species sensitivities alter the precise boundaries of the community flowering season [4,5,36]. The overall advance in LFD_com_ observed here may therefore reflect faster phenological development under interannual variation in hydrothermal conditions, whereas species removal mainly adjusted the magnitude of this advance by changing the composition and relative contributions of mid- and late-flowering species. Shortening of FD_com_ resulted from convergence of the beginning and end of the flowering season. LFD_com_ advanced over time in both the control and grass-removal treatments, and under grass removal, FFD_com_ was simultaneously delayed, producing a particularly pronounced temporal contraction of FD_com_.

In addition, forb removal, grass removal, and proportional thinning significantly reduced the temporal stability of both community-level first and last flowering dates, whereas dominant sedge removal had no significant effect. By contrast, the temporal stability of FD_com_ showed no significant response to species-removal treatments, despite reduced stability of FFD_com_ and LFD_com_. Because FD_com_ is defined as LFD_com_ − FFD_com_, positively correlated year-to-year shifts in the two flowering-season boundaries can partly cancel when their difference is taken, thereby reducing variation in flowering duration even when the two boundary dates vary substantially. Such coordinated shifts are plausible in alpine communities, where common environmental drivers can shift flowering onset and cessation in the same direction without substantially altering flowering duration [37]. Compensatory responses among species or flowering functional groups may provide additional biological buffering [33,38], although our data cannot distinguish this mechanism from the statistical consequence of constructing FD_com_ as the difference between two dates. For first flowering date, greater phenological divergence among flowering functional groups and increased community-wide dispersion induced by removal may have amplified interannual variation in the onset of the community flowering season and thereby reduced temporal stability [25,38]. By contrast, the decline in temporal stability of last flowering date was unrelated to changes in community-wide flowering-date dispersion, suggesting that it may have been jointly governed by other processes, including phenological shifts in late-flowering species and interannual environmental variation [29,36,39]. The absence of a significant stability decline following dominant-sedge removal suggests that compensatory dynamics among the remaining species or flowering functional groups may have maintained the interannual stability of community flowering-season boundaries [33,38]. Biodiversity–stability theory predicts that asynchronous responses among species or functional groups can buffer interannual variation through compensatory dynamics and thereby maintain the stability of community functioning [33,40,41]. Species loss may weaken temporal complementarity and response diversity among flowering functional groups, causing the beginning and end of the community flowering season to depend more strongly on a small number of remaining groups and consequently reducing temporal stability.

### 3.3. Species Loss Affected Flowering Synchrony and Temporal Stability by Increasing Phenological Divergence Among Flowering Functional Groups

Our results show that both declining species richness and increasing interannual variability in richness intensified phenological divergence among early-, mid-, and late-flowering functional groups. When species loss is concentrated within a particular portion of the flowering season, the otherwise continuous early–mid–late sequence is disrupted. At the same time, competitive release can induce phenological adjustments that differ in direction or magnitude among flowering functional groups, further increasing temporal separation among groups [9,31]. Lower temporal stability of species richness indicates greater interannual variation in the number of species in the community. When this variation is asynchronous among flowering functional groups, their mean flowering dates may also vary by different magnitudes among years, thereby increasing phenological divergence [25,33]. Higher soil temperature and greater soil available phosphorus were associated with greater divergence in first flowering date, but showed no significant relationship with divergence in last flowering date. First flowering is regulated by developmental thresholds and early-season environmental cues. Differences among flowering functional groups in temperature sensitivity and in nutrient acquisition and use strategies may therefore generate shifts in first flowering date that differ in direction or magnitude, increasing temporal separation among groups [36,37]. By contrast, last flowering date integrates processes operating throughout the reproductive period and is jointly influenced by flowering duration, resource depletion, and neighborhood competition in addition to environmental conditions. Divergence in last flowering date may therefore be driven more strongly by the reorganization of community composition and interspecific interactions following species loss [5,7]. Neither soil moisture nor soil available nitrogen had a significant effect, suggesting that, across the range observed in this study, these variables did not exert sufficiently strong asymmetric effects among flowering functional groups (Figure S2).

Phenological divergence among flowering functional groups was positively related to community-wide dispersion of both first and last flowering dates, indicating that separation in the mean phenological positions of the early-, mid-, and late-flowering groups propagated to the community scale. As temporal distances among groups increase, overall community flowering dates become more dispersed even if within-group variation changes little, thereby reducing community flowering-date synchrony [33,42,43]. Because both phenological divergence among flowering functional groups and community-wide flowering-date dispersion were calculated using standard deviations, their relationship partly reflects shared statistical structure. The result should therefore be interpreted as evidence that among-group divergence contributes strongly to overall community dispersion, rather than as independent causal evidence. Nevertheless, relationships between community-wide flowering date dispersion and temporal stability differed between first and last flowering dates. Greater dispersion of first flowering date was associated with lower temporal stability. As differences in first flowering among functional groups expand, community-level first flowering date may become more sensitive to interannual variation in the earliest-flowering species. When species loss further intensifies among-group phenological divergence, the species determining community first flowering may vary among years, allowing species-level phenological fluctuations to propagate more readily to the community scale and thereby increasing interannual variability and reducing temporal stability. By contrast, dispersion of community-level last flowering date was unrelated to its temporal stability, suggesting that synchrony and interannual variability in community last flowering are regulated by different processes. The temporal stability of last flowering date may depend more strongly on interannual changes in mid- and late-flowering species, their capacity to maintain flowering, and late-season environmental conditions, and therefore cannot be explained by phenological divergence among functional groups alone [11,39]. Future studies should disentangle the contributions of species turnover and shifts in relative abundance, which represent important dimensions of biodiversity change beyond species richness alone [44], from those of intraspecific phenological plasticity in shaping interannual variation in last flowering date, thereby clarifying the mechanisms underlying its temporal stability. Further studies across ecosystems differing in climate, species composition, and competitive environments would also help assess the generality of these patterns beyond this alpine meadow.

## 4. Materials and Methods

### 4.1. Data Source and Study System

A publicly available dataset from a long-term species-removal experiment was used in this study. The experiment was conducted at the Gannan Grassland Ecosystem National Observation and Research Station in Maqu County, China (101°52′ E, 33°39′ N; 3485–3542 m a.s.l.). The dataset is publicly available on Figshare (DOI: 10.6084/m9.figshare.29209094).

The study site is an alpine meadow with a mean annual temperature of 1.2 °C and mean annual precipitation of approximately 620 mm, based on meteorological records for 1975–2012; most precipitation falls between June and August [45]. Grazing has been excluded from the experimental site since 2007. The vegetation is characterized by high local species richness but low spatial turnover, with 20–35 species per 0.25 m^2^ and similar initial species composition among plots [46,47]. The plant community is dominated by the early-flowering sedge *Carex capillifolia*, together with diverse forbs and late-flowering grasses, and an overall advance in flowering phenology under recent warming has been observed [5].

### 4.2. Species-Removal Experiment

The species-removal experiment was established in May 2011 in a flat, homogeneous alpine meadow using a randomized block design [9]. Thirty-five permanent plots were arranged in seven blocks, each containing five treatments: (i) an unmanipulated control (Control); (ii) sedge removal (^×^Sedges_*K*.), in which the dominant sedge *Carex capillifolia* was removed to represent sedge loss associated with grazing, warming, and nitrogen enrichment [48,49]; (iii) forb removal (^×^Forbs), in which all forb species were removed to represent the decline in forb diversity under nitrogen enrichment [48]; (iv) grass removal (^×^Grasses), in which grasses were removed to simulate grass loss under overgrazing and drought [2,4]; and (v) proportional thinning across plant functional groups (^×^Thinning), in which 1–8 species from sedges, forbs, and grasses were removed to mimic stochastic species loss or non-selective grazing while maintaining overall community structure and competitive hierarchy [47,50]. Target species were manually clipped twice per week during May and June from 2011 to 2016, with any newly germinated seedlings or regenerated individuals of the target species also removed during routine plot maintenance; by 2016, their regrowth abundance had fallen below 1%. Flowering phenology of the remaining species was subsequently monitored from May 2017 onwards at population levels.

### 4.3. Flowering Phenology Observations

For the present analysis, we used population-level flowering phenology records from 2017 to 2023 in the archived dataset. Observations were conducted weekly from May to August for 13 focal species spanning the dominant plant functional groups and flowering periods of the community. For each species in each quadrat, first flowering date (FFD_pop_) was defined as the day of year on which the cumulative number of observed flowers or florets reached 10% of the seasonal total, and last flowering date (LFD_pop_) as the day of year on which 90% of flowers had senesced [4]. Flowering duration (FD_pop_) was calculated as LFD_pop_ − FFD_pop_. Community-level flowering phenology was calculated for each treatment plot by pooling flowering records across all observed species. The dates on which cumulative community flowering reached 10% and 90% were defined as FFD_com_ and LFD_com_, respectively, and FD_com_ was calculated as LFD_com_ − FFD_com_ [13].

### 4.4. Soil Environmental Measurements

To characterize soil environmental conditions, soil temperature (ST) and soil moisture (SM) at a depth of 5 cm (the primary rooting zone) were monitored automatically at 15 min intervals using an EM50 Data Collection System (Decagon Devices, Inc., Pullman, WA, USA) from May to September each year [4,13]. Annual growing-season means of ST and SM were used in subsequent analyses. In August 2022, five soil cores (0–20 cm depth) were collected from each plot and combined into a composite sample. Soil ammonium (NH_4_^+^) and nitrate (NO_3_^−^) were extracted with 2 M KCl, and soil available phosphorus (AP) was extracted with 0.5 M NaHCO_3_. Concentrations were determined using an automated discrete analyzer (SmartChem 450, AMS Alliance, Rome, Italy), and soil available nitrogen (AN) was calculated as the sum of NH_4_^+^ and NO_3_^−^ [50].

### 4.5. Statistical Analyses

All statistical analyses were performed in R version 4.5.0 [51]. Linear mixed-effects models were fitted with the *lme4* package using restricted maximum likelihood (REML; [52]), and figures were produced with the *ggplot2* package [53].

The 13 focal species were assigned to early-, mid-, or late-flowering functional groups based on their flowering timing within the growing season (Table S1). Species flowering before the beginning of June were classified as early-flowering species, those flowering from June to July as mid-flowering species, and those flowering from August onward as late-flowering species. To ensure comparability among treatments, analyses of the effects of species removal on the flowering phenology of remaining species included only species that occurred in both the control and the corresponding removal treatment, thereby minimizing bias arising from differences in species composition. We analyzed three flowering phenology metrics: population-level first flowering date (FFD_pop_), last flowering date (LFD_pop_), and flowering duration (FD_pop_). For each metric, we fitted a linear mixed-effects model to evaluate the effects of removal treatments on species phenology within each flowering functional group (Figure 2). The model structure was Y ~ treatment + (1|year) + (1|block) + (1|species), where Y denotes FFD_pop_, LFD_pop_, or FD_pop_; *treatment* was included as a fixed effect; and *year*, *block*, and *species* were included as random effects to account for interannual variation, spatial block effects, and intrinsic differences among species. Models were fitted separately for the early-, mid-, and late-flowering functional groups. Because the nine models generated 27 treatment-effect comparisons in Figure 2, these *p*-values were jointly adjusted for multiple testing using the Benjamini–Hochberg false discovery rate procedure. This approach was chosen to control the expected proportion of false discoveries across the set of related phenological comparisons while maintaining adequate statistical power. Statistical significance in Figure 2 was determined using the resulting BH-adjusted *p*-values.

To test the effects of species loss on community flowering-date synchrony (Figure 3a,b), we calculated the standard deviations of first and last flowering dates across all species within each year, treatment, and block, denoted FFD_sd and LFD_sd, respectively. These standard deviations quantify the dispersion of community flowering dates and are inversely related to community flowering-date synchrony. Larger standard deviations indicate greater dispersion and therefore lower flowering-date synchrony, whereas smaller values indicate greater temporal concentration and higher synchrony. They describe the temporal concentration of flowering onset and cessation events rather than pairwise overlap across complete flowering periods. Separate linear mixed-effects models were fitted for the two metrics using FFD_sd (or LFD_sd) ~ treatment + (1|year) + (1|block), with *treatment* as a fixed effect and *year* and *block* as random effects to account for interannual and spatial variation. Larger standard deviations indicate greater dispersion of flowering dates and lower community flowering-date synchrony, whereas smaller values indicate more concentrated flowering dates and higher synchrony [4,43]. Further, for each removal treatment, we constructed the null model independently within each year × block combination using the corresponding control community. In each randomization, the number of species retained in the randomized control community was set equal to the observed species richness of the corresponding removal treatment in the same year and block; equivalently, the number of species randomly removed from the control equaled the difference in realized species richness between the control and treatment communities. Only species actually present in the corresponding control plot-year and with non-missing FFD and LFD records were eligible for randomization. Species were sampled without replacement within each randomization, and this procedure was repeated 500 times for each treatment × year × block combination. Changes in FFD_sd and LFD_sd relative to control were calculated as the treatment value minus the corresponding control value, and the same calculation was applied to each randomized community. The mean and 95% randomization interval of the null distributions were then compared with the observed values (Figure 3c,d). Observed values falling outside the 95% randomization interval indicated that the effects of species removal on flowering synchrony differed significantly from those expected under random species loss.

We then examined how species-removal treatments affected interannual trends and temporal stability in community flowering phenology. Community-level first flowering date, last flowering date, and flowering duration (FFD_com_, LFD_com_, and FD_com_, respectively) were calculated for each year, treatment, and block. Points in Figure 4a–c show treatment-year means (points) ± SE (error bars) across blocks; fitted lines are shown only for significant within-treatment interannual trends (*p* < 0.05). The significance tests for all within-treatment interannual trends are reported in Table S2. To test temporal trends and differences in trends among treatments, year was centered on 2020 (year_c = year − 2020), and a linear mixed-effects model was fitted as Y ~ year_c × treatment + (1|block), with centered *year*, *treatment*, and their interaction as fixed effects and *block* as a random effect. Temporal stability of FFD_com_, LFD_com_, and FD_com_ was then calculated for each treatment within each block across 2017–2023 as the ratio of the multiyear mean to the interannual standard deviation (μ/σ). Using control within the same block as the reference, we calculated the log response ratio of temporal stability for each removal treatment as lnRR = ln(stability_treatment_/stability_Control_). Bars represent the mean ± SE of block-level lnRR values for each treatment. One-sample *t*-tests were used to determine whether lnRR differed significantly from zero. Values of lnRR < 0 indicate lower temporal stability than control, whereas lnRR > 0 indicates higher temporal stability (Figure 4d–f).

We further evaluated the relative roles of species richness and soil environmental changes in driving phenological divergence among flowering functional groups. We calculated the mean first and last flowering dates of the early-, mid-, and late-flowering functional groups for each year, treatment, and block. Phenological divergence among groups was quantified as the standard deviation of the three group means, with larger values indicating greater phenological separation. Separate multiple linear regression models were fitted for first and last flowering dates. The response variable was the change in phenological divergence relative to control, and predictors were species richness loss, the temporal stability of species richness, soil temperature and its temporal stability, soil moisture and its temporal stability, soil available nitrogen, and soil available phosphorus. Temporal stability of species richness, soil temperature, and soil moisture was calculated across 2017–2023 as the multiyear mean divided by the interannual standard deviation (μ/σ). Before modeling, response variables and continuous predictors were *z*-standardized. Models were fitted by ordinary least squares, and the relative effects of predictors were evaluated using standardized regression coefficients (*β*) and their 95% confidence intervals (Figure 5a,d). We also quantified community-wide dispersion of flowering dates as the standard deviation of first or last flowering dates across all species within each year, treatment, and block. To compare treatment effects, we calculated the multiyear means of phenological divergence among flowering functional groups and community-wide flowering-date dispersion for each treatment and block across 2017–2023, and then calculated their changes relative to control within the same block (ΔSD). Larger ΔSD values indicate stronger increases in phenological divergence among flowering functional groups or in community-wide dispersion of flowering dates caused by the removal treatment. Finally, simple linear regressions were used to test the relationships between changes in phenological divergence among flowering functional groups and changes in community-wide flowering-date dispersion (Figure 5b,e), and between changes in community-wide flowering-date dispersion and the relative temporal stability of first or last flowering date (Figure 5c,f).

## 5. Conclusions

Our study suggests that plant species loss can modestly alter flowering phenology in an alpine meadow by inducing divergent responses among early-, mid-, and late-flowering functional groups. These shifts were accompanied by non-random changes in community flowering-date synchrony—forb removal decreased synchrony, whereas grass and sedge removal increased synchrony—and by reduced temporal stability of both first and last flowering dates. Phenological divergence among functional groups was a strong predictor of community-wide flowering-date dispersion, while greater dispersion corresponded to lower temporal stability for first flowering date but not for last flowering date.

These patterns may have important implications for pollination and plant reproduction. Changes in flowering synchrony could alter the temporal continuity of floral resources and flowering overlap among species, with potential consequences for pollinator visitation, outcrossing, and seed set, while greater interannual variability may reduce the predictability of reproductive opportunities and influence longer-term community dynamics. From a conservation perspective, our findings suggest that the phenological identity of species loss deserves consideration, because the consequences of species removal depended on which flowering functional group was affected. Maintaining representation across early-, mid-, and late-flowering groups may therefore help sustain the temporal continuity of floral resources in alpine communities. Soil temperature and phosphorus were also related to phenological divergence, suggesting that environmental conditions may further shape these patterns. Because our study was conducted at a single alpine meadow site, cross-ecosystem comparisons are needed to determine the generality of these relationships. Overall, our findings highlight the importance of conserving both species richness and phenological diversity to maintain the temporal organization and functioning of alpine plant communities.

## Data Availability

The data analyzed in this study were obtained from a publicly available dataset deposited in Figshare and can be accessed at https://doi.org/10.6084/m9.figshare.29209094 [54].

## Supplementary Materials

The following supporting information can be downloaded at: https://www.mdpi.com/article/10.3390/plants15182822/s1, Table S1: List of the focal species and their flowering and plant functional groups; Table S2: Temporal trends in community-level flowering phenology under different treatments from 2017 to 2023; Figure S1: Effects of species richness, soil temperature, and soil available phosphorus on the flowering phenology of different flowering functional groups; Figure S2: Effects of soil moisture and soil available nitrogen on the flowering phenology of different flowering functional groups.
