# Supplementary material for: Differential Responses of Flowering Functional Groups to Species Loss Altered Community Flowering-Date Synchrony and Reduced Temporal Stability in an Alpine Meadow
Source: Plants (Basel). 2026 Sep 14;15(18):2822. doi: 10.3390/plants15182822 (PMC13610139; doi:10.3390/plants15182822)
Supplement: Supplementary file 1 [file plants-15-02822-s001.zip › plants-4502179-supplementary.pdf]

Differential responses of flowering functional groups to species loss altered community flowering-date synchrony and reduced temporal stability in an alpine meadow

Supporting Information

List of supporting information

Table S1. List of the focal species and their flowering and plant functional groups.

Table S2. Temporal trends in community-level flowering phenology under different treatments from 2017 to 2023.

Figure S1. Effects of species richness, soil temperature, and soil available phosphorus on the flowering phenology of different flowering functional groups.

Figure S2. Effects of soil moisture and soil available nitrogen on the flowering phenology of different flowering functional groups.

Table S1 List of the focal species and their flowering and plant functional groups

Species	Abbreviation	Flowering functional group	Functional group
Kobresia capillifolia	Kc	Early	sedges
Carex atrofusca	Ca	Early	sedges
Anemone obtusiloba	Ao	Early	forbs
Thalictrum alpinum	Ta	Early	forbs
Euphorbia esula	Ee	Mid	forbs
Rumex patientia	Rp	Mid	forbs
Ranunculus tanguticus	Rt	Mid	forbs
Potentilla fragarioides	Pf	Mid	forbs
Poa poophagorum	Pp	Mid	grasses
Elymus nutans	En	Late	grasses
Koeleria macrantha	Km	Late	grasses
Stipa aliena	Sa	Late	grasses
Delphinium kamaonense	Dk	Late	forbs

Note: The 13 focal species listed in the table were monitored in this study and collectively spanned the entire flowering season and represented three plant functional groups. Based on their flowering timing during the growing season, the species were classified into three flowering functional groups: species flowering before the beginning of June were classified as early-flowering species (Early), those flowering from June to July as mid-flowering species (Mid), and those flowering from August onward as late-flowering species (Late).

Table S2. Temporal trends in community-level flowering phenology under different treatments from 2017 to 2023.

Treatment	Annual trend (slope ± SE, days year^{-1})	*p*-value
**First flowering date (FFD_{com})**		
Control	0.35 ± 0.43	0.42
×Thinnning	-0.63 ± 0.44	0.15
×Sedges_*K.*	-0.59 ± 0.43	0.17
×Grasses	**1.08 ± 0.45**	**0.02**
×Forbs	**-0.84 ± 0.43**	**0.05**
**Last flowering date (LFD_{com})**		
Control	**-1.28 ± 0.41**	**<0.01**
×Thinnning	-0.51 ± 0.42	0.22
×Sedges_*K.*	**-1.57 ± 0.41**	**<0.01**
×Grasses	**-1.69 ± 0.43**	**<0.01**
×Forbs	-0.34 ± 0.41	0.41
**Flowering duration (FD_{com})**		
Control	**-1.62 ± 0.51**	**<0.01**
×Thinnning	0.12 ± 0.52	0.82
×Sedges_*K.*	**-0.99 ± 0.51**	**0.05**
×Grasses	**-2.77 ± 0.54**	**<0.01**
×Forbs	0.50 ± 0.52	0.33

Tabular values show treatment-specific annual trends (estimated slopes ± SE) from linear mixed-effects models fitted as response ~ $year_c$ × treatment + (1 | block) using maximum likelihood, where $year_c$ = year − 2020. The *p* values are the original two-sided values returned by emmeans::emtrends. Significant trends ($p < 0.05$) are highlighted in bold and are the only treatment-specific regression lines shown in Figure 4.

Figure S1. Effects of species richness, soil temperature, and soil available phosphorus on the flowering phenology of different flowering functional groups.

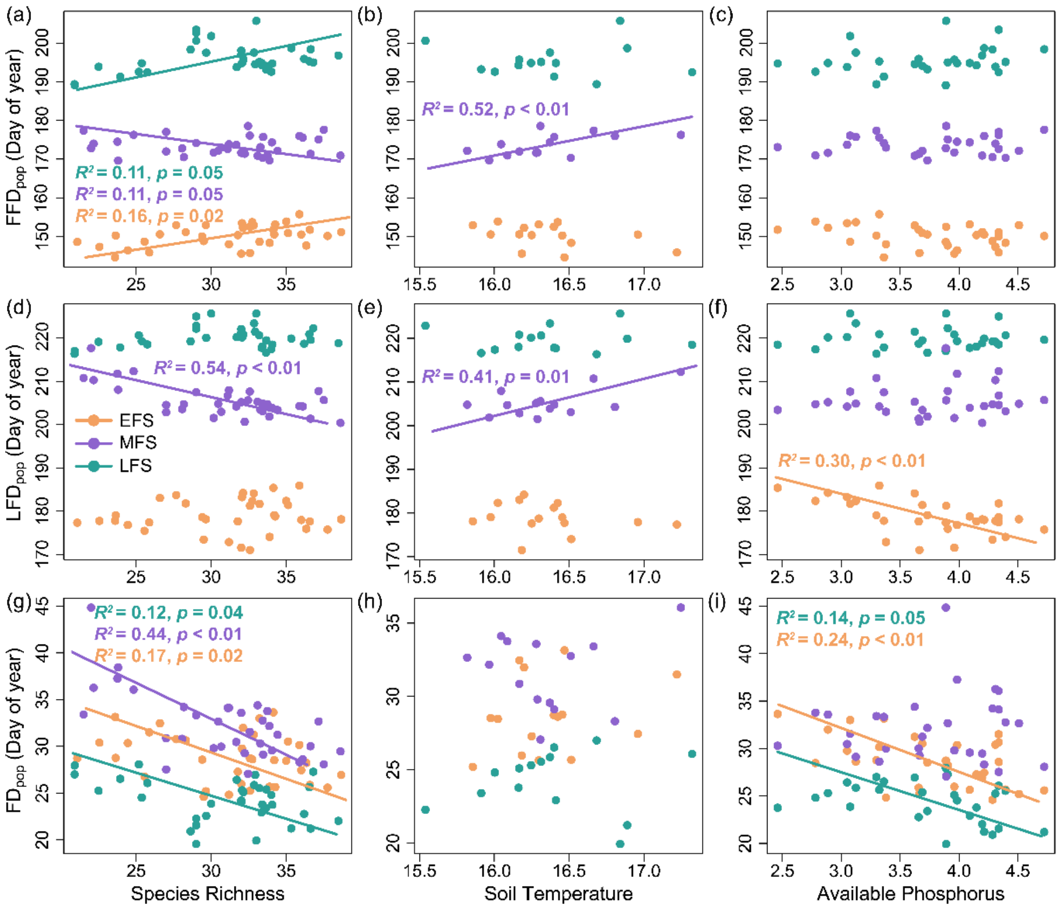

Relationships of species richness, soil temperature, and soil available phosphorus with first flowering date, last flowering date, and flowering duration were analyzed using linear mixed-effects models. Panels (a–c) show the relationships of first flowering date with species richness, soil temperature, and soil available phosphorus, respectively; panels (d–f) show the corresponding relationships for last flowering date; and panels (g–i) show those for flowering duration. Colors denote flowering functional groups: orange, early-flowering species; purple, mid-flowering species; and blue-green, late-flowering species. Points represent observations, and lines represent trends fitted by the linear mixed-effects models. Solid lines indicate significant relationships ($p < 0.05$), whereas dashed lines indicate non-significant relationships.

Figure S2. Effects of soil moisture and soil available nitrogen on the flowering phenology of different flowering functional groups.

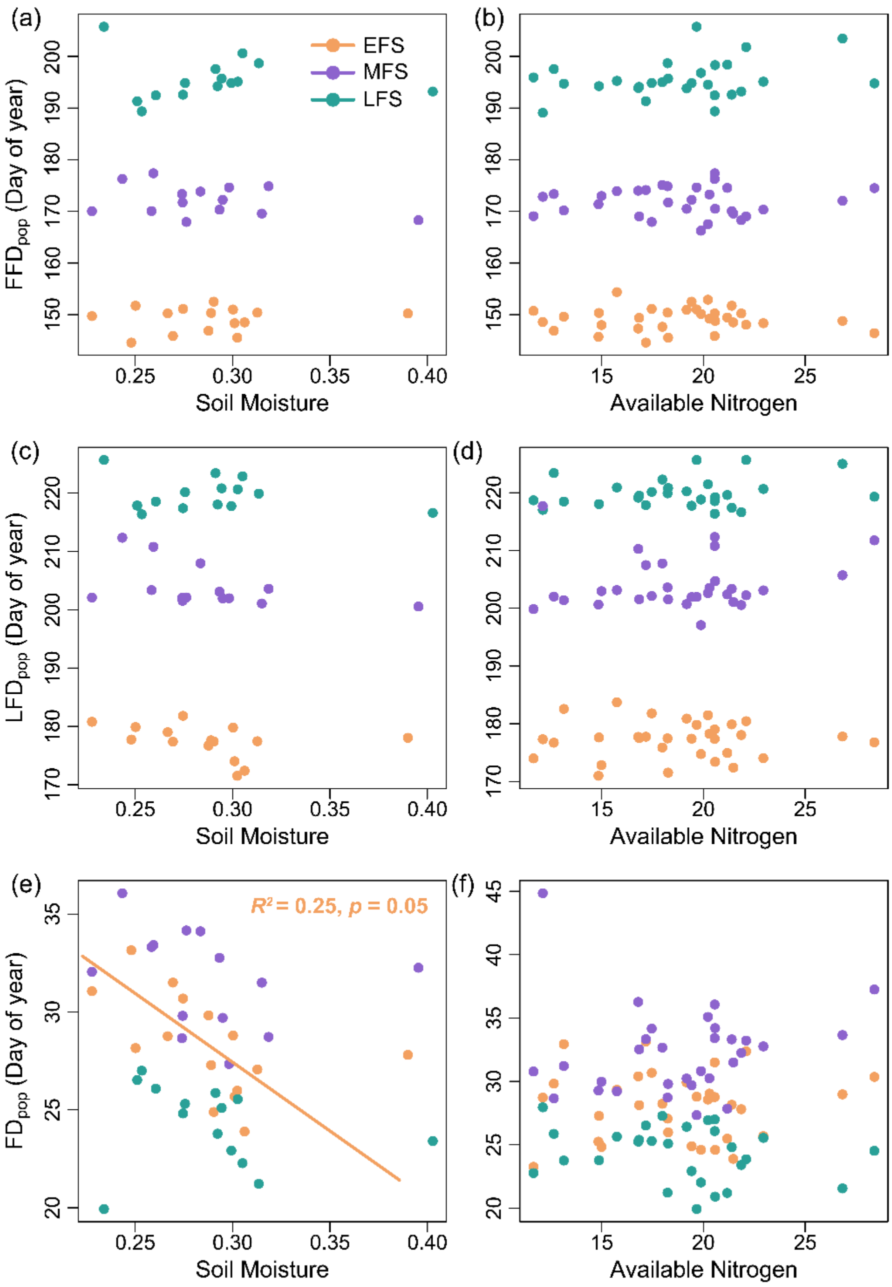

Relationships of soil moisture and soil available nitrogen with first flowering date, last flowering date, and flowering duration were analyzed using linear mixed-effects models. Panels (a–b) show the relationships of first flowering date with soil moisture and soil available nitrogen, respectively; panels (c–d) show the corresponding relationships for last flowering date; and panels (e–f) show those for flowering duration. Colors denote flowering functional groups: orange, early-flowering species; purple, mid-flowering species; and blue-green, late-flowering species. Points represent observations, and lines represent trends fitted by the linear mixed-effects models. Solid lines indicate significant relationships ($p < 0.05$), whereas dashed lines indicate non-significant relationships.
